# Annotations

**Published:** 1907-04-06

**Authors:** 


### Annotations.

### The Carnegie Trust.

The Carnegie Trust for the Universities of Scotland has now been in operation for over five years, and the first quinquennial appropriation of grants expires in December next. It is of interest to note some of the work of the Trust during this period. The total income for the five years has been nearly £543,000. Of this no less than £242,000 has been advanced to students for the payment of class fees, whilst grants amounting to £175,000 have been made to the several Universities for buildings, permanent equipment, increase of teaching staff, libraries, research, etc. The value of these grants may be appreciated from a statement which has just appeared in a report issued to the General Council of the University of Glasgow. Here, during the period in question, a sum of £16,000 has been allotted to the extension of the Natural Philosophy department so long and illustriously associated with the name of Lord Kelvin; a similar amount has been paid towards the provision of a physiological laboratory; a contribution of £7,500 has been advanced towards the endowment of a chair of geology; and a sum of £4,000 has been given to the University library. The other Universities have shared in an equally good fortune, and some £18,000 has been spent on research scholarships and fellowships. The friends of education everywhere may well join the immediate beneficiaries in gratitude to Mr. Carnegie for his enlightened generosity. Whether the £242,000 advanced in payment of students' fees is an equally wise and beneficial expenditure may be open to question. This particular responsibility is one which has evidently caused the trustees some anxiety, and they have recently increased the stringency of the conditions under which such grants are made. It is of interest to note that a sum of nearly £2,000 has been repaid by students who were formerly benefited by the Trust.

### Société de l'Entente Cordiale Médicale.

This society is the outcome of the visit of the French physicians to London in October 1904, and of the subsequent visit of a number of English physicians to Paris. It now numbers some six hundred members, and among the English Presidents are Sir Wm. Broadbent and Sir Dyce Duckworth, while Mr. Leonard Bidwell is the Honorary Secretary. The object of the society is announced in its name, and to promote this object a series of meetings, to take place alternately in England and France, has been arranged. It was expected that Sir Wm. Broadbent would have lectured in Paris in January last, but his illness unfortunately prevented this. In the immediate future it is anticipated that Dr. Pierre Marie will deliver a lecture in London. He has announced as his subject "Aphasia," and in view of his recently published views it is certain that his lecture will attract the greatest attention and interest. Amidst the certain facts of cerebral localisation the function and value of Broca's lobe have long occupied a prominent, and apparently, a secure, place. Dr. Marie has thrown the whole question of cerebral localisation in relation to speech function into the melting-pot, and so fundamental an inquiry as he has started may be expected to produce widespread disturbances. Certainly many will be anxious to hear his arguments at first hand. In connection with the society mentioned above we may allude once again to the *Paris Medical Journal*. This has just celebrated its first anniversary, and we gladly offer to our contemporary our cordial congratulations and goodwill. All English-speaking physicians who wish to keep in touch with French thought and teaching on medical and surgical questions cannot do better than consult our young contemporary's pages.

### Tennis Elbow.

Nearly every form of sport is liable to give rise to some special surgical lesion, generally involving some particular region of the skeleto-muscular system, and taking the form either of a fracture of a bone or a tearing of some muscle or ligament. In most cases the lesion is definite and obvious, but in the case of the so-called tennis elbow the exact nature of the injury is somewhat obscure. It is highly probable that the lesion is not quite the same in all cases, although there are certain signs common to all. In most cases there is definite tenderness on pressure about the external condyle of the humerus, especially on the posterior aspect, and acute pain is caused by any forcible and complete extension of the elbow. In some cases this pain is felt most sharply when the arm is extended and at the same time supinated, as occurs when a low ball is struck in tennis. In these cases it is probable that the injury has been inflicted in the effort to take such a ball, and, according to Clado, the lesion consists in the tearing of some of the fibres of the supinator brevis. According to Preiser, of Hamburg, however, there is no rupture of muscular fibre, but a tearing of the humero-radial ligamentous capsule, owing to a simultaneous forcible contraction of the supinator brevis and brachialis anticus muscles, and resulting in a chronic synovitis with effusion. In other cases, again, the injury appears to be caused by a forcible back-handed stroke, and it is this stroke in particular which causes most pain, and may give rise to a recurrence of the symptoms after they have subsided. In these cases the forearm is extended and pronated, and the extensor muscles of the arm and forearm are chiefly involved. Considering the position of the injury, it seems probable that in these cases the injury consists in a tearing of muscular fibres either of the little anconeus muscle or of the extensor muscles attached to the external condyle. It is certainly unusual to find any definite swelling of the part, such as one should get in the case of a synovitis with effusion of the radial articulation, so that the evidence seems in favour of a rupture of certain muscular fibres, which may vary according to the way in which the injury is inflicted. Tennis elbow is a troublesome condition, very intractable to treatment, and liable to recur even after prolonged rest.

